# Advances in Optimized and Safe Path Planning of Marine Autonomous Surface Vehicles: A Review

**DOI:** 10.3390/s26113445

**Published:** 2026-05-29

**Authors:** Lirong Kou, Xiaoyang Gao

**Affiliations:** School of Maritime Economics and Management, Dalian Maritime University, Dalian 116026, China

**Keywords:** autonomous surface vehicles, path planning, collision avoidance for safety, optimization

## Abstract

With the rapid development of intelligent shipping and the autonomy of marine engineering equipment, numerous studies have focused on the advancement of Autonomous Surface Vehicles (ASVs). As a fundamental component of ASV automation systems, path planning directly determines the safety and economy of ship navigation. This paper systematically reviews recent research progress in ASV path planning. First, five key issues are identified for ASV path planning: navigation environment, environment modeling, ship motion model, collision avoidance for safety, and optimization. Second, existing algorithms are classified into four categories: graph search-based, sampling-based, numerical optimization-based, and artificial intelligence-based. The improvement directions and application scenarios of each category are elaborated. Finally, the four types of algorithms are evaluated against three indicators: path quality, scalability and extensibility, and algorithm performance. Analysis of the reviewed literature shows that traditional graph search and sampling algorithms perform well in various aspects under static environments, but are insufficient in adapting to multiple constraints and generalizing to different environments. In contrast, artificial intelligence algorithms represented by deep reinforcement learning exhibit significant advantages in dynamic collision avoidance decision-making, multi-agent coordination, and environmental generalization, and have become the mainstream direction of current research. This paper summarizes the existing challenges in safety and optimization in current ASV path planning research and prospects future development directions.

## 1. Introduction

In recent years, intelligent shipping and autonomous engineering equipment have become core directions in the development of marine technology. Highly autonomous ships, such as uncrewed surface vehicles (USVs), have become a research hotspot in marine engineering [1]. In previous studies, uncrewed ground vehicles and uncrewed aerial vehicles have received extensive attention. Uncrewed ground vehicles (UGVs) have been widely used in logistics, warehousing, military affairs, public services, and other fields [2,3,4]; applications of uncrewed aerial vehicles (UAVs) in agriculture, transportation, surveillance, emergency response, and other fields have promoted the intelligent development of related industries [5,6]. USVs also show advantages in safety, comfort and flexibility in both military and civilian applications [7,8]. Compared with traditional manual operation, highly autonomous uncrewed equipment presents remarkable advantages in cost and efficiency, and reduces casualties in high-risk operating environments. Unlike ground and aerial vehicles, ships navigate in strongly disturbed and unstructured marine environments, which imposes stricter requirements on path safety, compliance, and robustness. Furthermore, with the development of the shipping industry and the increasing number of vessels, higher demands are placed on the precision of automatic operation and adaptability to complex environments.

Path planning is the foundation of ship automation systems, encompassing environment perception, motion constraint adaptation, and collision avoidance decision-making, realizing cross-domain technological integration. The traditional path planning problem is described as: “Where am I? Where am I going? How do I get there?” [9]. This framework applies to simple path planning tasks, but to some extent ignores the impacts of complex factors such as environment and dynamics on path planning. On this basis, modern path planning must additionally consider the influence of complex navigation environments and the ship’s own actuation characteristics on ship motion. Therefore, Zhou et al. [10] extended the problem to four questions: “What’s my situation?”, “Where can I go?”, “Where should I go?”, and “How should I do to get there?”. However, current research demands more than basic path feasibility—it requires optimization, necessitating a fifth critical question: “How can I arrive more efficiently?”.

This review focuses on ASV path planning algorithms, emphasizing their comprehensive performance and safe collision avoidance. The selected literature primarily comprises novel studies on vessel path planning published in leading journals such as IEEE Transactions on Intelligent Transportation Systems and Ocean Engineering, covering the period from 2025 to 2026. We also include several works originally developed for other intelligent agents that offer valuable insights for ASV path planning, while excluding repetitive studies and those with limited relevance. By decomposing the path planning task into detailed subproblems and conducting a multi-dimensional comparative analysis of representative algorithms, this paper delineates the core strengths and limitations of each category. Compared with existing reviews, this work aims to provide a more comprehensive and forward-looking perspective on ASV path planning, thereby contributing to enhanced navigation safety and path quality.

Section 2 clarifies the formal problem definition and core constraints of ASV path planning. Section 3 categorizes state-of-the-art algorithms and analyzes their underlying principles, key improvements, and application scenarios. Section 4 conducts a systematic comparative analysis of different algorithms across three dimensions. Finally, Section 5 summarizes the remaining limitations of current approaches and outlines promising future research directions.

## 2. Preliminaries and Problem Formulation

Zhou et al. [10] divided the development of path planning into three stages: route planning, trajectory planning, and motion planning. This progression can be summarized as macro-level design to micro-level control, and from simple constraints to complex constraints. In the current research, for a complete and practically applicable path planning framework, the five fundamental questions—“What’s my situation?”, “Where can I go?”, “Where should I go?”, “How should I do to get there?”, and “How can I arrive more efficiently?”—can be concretized into five sub-problems: navigation environment, environment modeling method, ship motion model, collision avoidance for safety, and path optimization.

**Figure 1:**
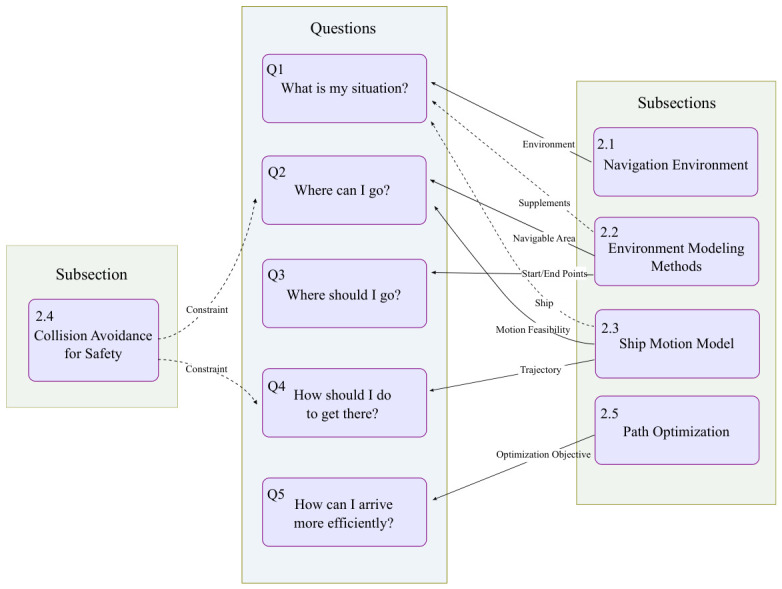
Mapping between core questions and subsections.

Specifically, the navigation environment answers Q1 by providing the basic environmental information. The environment modeling method addresses Q2 (in terms of navigable regions) and Q3, while also supplementing Q1 with additional environmental details. The ship motion model directly answers Q2 (in terms of dynamic maneuverability constraints) and Q4, and further elaborates on the vessel’s state. Collision avoidance for safety serves as a constraint spanning both Q2 and Q4. The purpose of path optimization is to answer the final question. The correspondence is illustrated in Figure 1.

### 2.1. Navigation Environment

The marine navigation environment directly determines the constraints and optimization objectives of path planning. According to geographical characteristics and environmental influencing factors, marine navigation environments can be generally categorized into three types: confined waters, open waters, and polar waters.

#### 2.1.1. Confined Waters

Confined waters refer to navigable areas with severe maneuvering constraints, including inland waterways, bridge areas, and port fairways. These waters are characterized by high traffic density and complex hydrological characteristics. The primary environmental factors for path planning are tidal currents and wind currents. Local variations in current vectors and direction are common, driven by submarine topography and infrastructure such as bridge piers. In contrast, wind effects exhibit relatively stable spatial distribution and intensity. Additionally, confined waters feature dense static obstacles and frequent vessel encounters, requiring path planning to simultaneously satisfy multiple constraints: navigable channel boundaries, environmental disturbances, and COLREGs-compliant collision avoidance.

#### 2.1.2. Open Waters

Open waters mainly refer to navigation areas without obvious spatial restrictions, such as the open ocean. These waters have low traffic density. Important environmental factors for path planning include ocean currents and wind fields, both of which are influenced by macro-scale climatological systems. Vessels typically avoid predictable large-scale extreme weather events (e.g., typhoons). When navigating near islands or reefs, additional under keel clearance must be considered.

#### 2.1.3. Polar Waters

Polar waters are a unique type of navigation environment, mainly including the Arctic and Antarctic waters. In these regions, the impact of sea ice on vessel navigation must also be considered. The influence of ice varies significantly with the type of vessel. For icebreakers, sea ice is treated as either an obstacle or a high-resistance zone. For other vessels, it is regarded as an impassable obstacle. Notably, sea ice drift is driven by ocean currents; path planning must consider both the spatial distribution and time-varying characteristics of sea ice, while comprehensively balancing voyage distance and fuel consumption.

### 2.2. Environment Modeling Methods

In environment modeling, the selection of start and end points and the map modeling approach must be considered. In global path planning, the start and end points are usually ports or specified task waypoints, whose positions are globally known and remain unchanged for a long time. In local path planning, the start point is the current position of the vessel or its position at a future moment; the end point is dynamic. For fixed tasks, the end point is represented as a target area. For dynamic tasks, the end point is readjusted according to real-time task assignment.

**Figure 2:**
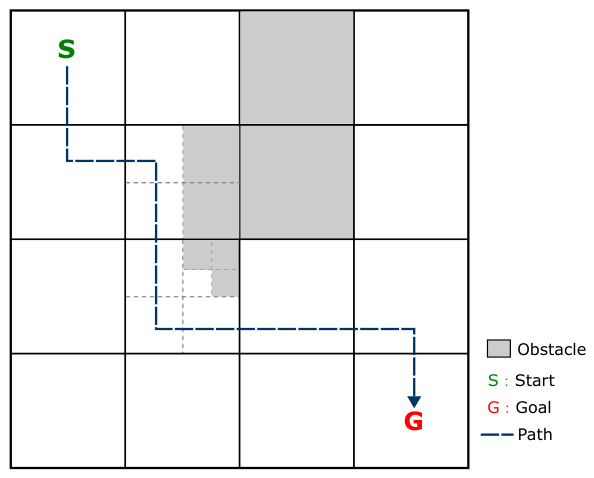
Grid map and quadtree decomposition.

Map modeling methods are mainly divided into two categories: grid-based maps and continuous maps. A grid-based map divides the map into multiple small cells, which can be represented by a two-dimensional array or matrix. To improve the accuracy of grid-based maps, the quadtree decomposition method is used. The quadtree method recursively decomposes non-homogeneous grids into four smaller grids each time, and stops decomposition when the environment within a grid is homogeneous. The quadtree method retains the detailed environmental changes in the grid map to the greatest extent and reduces redundant calculations caused by uniform map refinement. The schematic diagram of the grid map and quadtree decomposition is shown in Figure 2.

**Figure 3:**
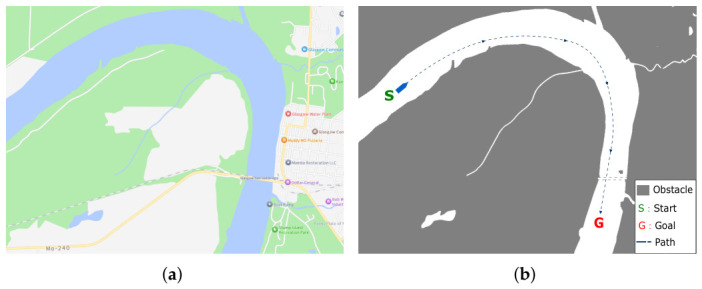
Example of continuous map modeling: (**a**) Real-world map. (**b**) Continuous map model.

Continuous maps are based on real maps and use continuous functions or vector models to describe information such as ocean currents and obstacle boundaries. Continuous maps can accurately represent the continuous distribution and gradual variation characteristics of ocean currents and wind fields, which are more consistent with real navigation scenarios. However, continuous maps significantly increase the computational load of path planning and require high hardware performance of shipborne equipment. On the other hand, such maps have poor algorithm compatibility, as traditional algorithms can hardly be directly applied, and they are mainly suitable for numerical optimization algorithms. The schematic diagram of continuous map modeling is shown in Figure 3.

### 2.3. Ship Motion Model

The ship motion model is the key to connecting the path planning module and the ship control module. Current path planning studies mainly focus on the position and motion of the ship, so a 3 Degrees of Freedom (3-DOF) model composed of surge, sway, and yaw is adopted to describe ship motion from the 6 DOFs of roll, pitch, yaw, sway, surge, and heave. The other three DOFs have relatively little influence on ship position and navigation but are closely related to comfort, and are therefore usually neglected in path planning. The 3-DOF model uses a dual-coordinate system, namely the earth- and body-fixed frames. The earth-fixed reference frame is adopted to characterize the vessel’s absolute position and heading in the global navigation space, whereas the body-fixed frame quantifies its relative linear velocities and angular velocity. Considering the coupling effects of hydrodynamic damping, environmental disturbances, and control inputs, the 3-DOF underactuated kinematic–dynamic model [11,12] can be expressed as:

(1) $$\left\{\begin{array}{cc} \dot{\eta } & =R(\psi )\nu \\ \dot{\nu } & =M^{-1}\left(f\left(\nu \right)+\tau_{w}+\tau \right) \end{array}\right.$$

where $\eta ={\left[p^{T},\psi \right]}^{T}$ denotes the vessel’s position p and heading angle ψ in the global coordinate system; $\nu ={\left[v^{T},r\right]}^{T}$ represents the surge and sway velocities $v={\left[u,v\right]}^{T}$ and the yaw rate *r*. $R\left(\psi \right)=\mathrm{diag}(R_{0}\left(\psi \right),1)$ is the rotation matrix with $R_{0}\left(\psi \right)=\left[\begin{array}{cc} \cos\psi & -\sin\psi \\ \sin\psi & \cos\psi \end{array}\right]$, $M=\left[\begin{array}{ccc} m_{11} & 0 & 0\\ 0 & m_{22} & m_{23}\\ 0 & m_{32} & m_{33} \end{array}\right]$ is the generalized positive-definite inertia matrix. $\tau ={\left[\tau_{u},0,\tau_{r}\right]}^{T}\in R^{3}$ represents the underactuated control input, $\tau_{w}\in R^{3}$ is the external disturbance term, and $f\left(\nu \right)\in R^{3}$ is the internal dynamic term.

The above model treats the vessel as a whole, whereas the Maneuvering Modeling Group (MMG) model decomposes the total hydrodynamic forces and moments into contributions from the hull, propeller, and rudder. Accordingly, the total surge force *X*, sway force *Y*, and yaw moment $N_{m}$ in the body-fixed frame are expressed as [13]:

(2) $$\left\{\begin{array}{c} X=X_{H}+X_{R}+X_{P}\\ Y=Y_{H}+Y_{R}\\ N_{m}=N_{H}+N_{R} \end{array}\right.$$

Here, subscripts *H*, *P* and *R* means hull, propeller, and rudder, respectively. The MMG-based 3-DOF model balances modeling accuracy and computational complexity, enabling path planning algorithms to seamlessly interface with the vessel’s trajectory tracking and motion control algorithms.

### 2.4. Collision Avoidance for Safety

Collision avoidance is the core safety constraint in path planning. Autonomous collision avoidance for vessels requires consideration of obstacle types, compliance with the International Regulations for Preventing Collisions at Sea (COLREGs), ship domain definition, and collision risk quantification.

**Figure 4:**
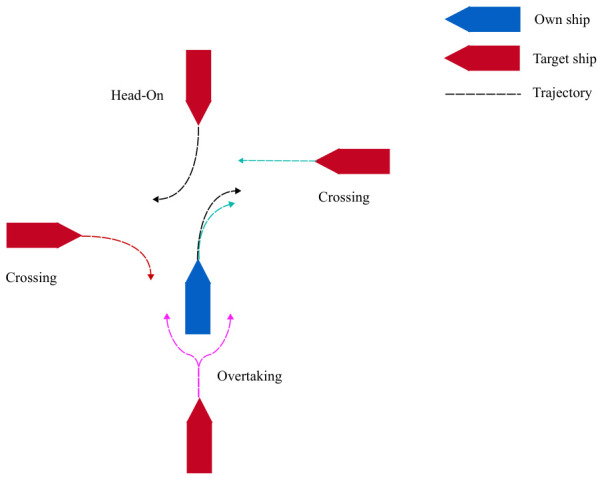
Schematic of COLREGs rules.

Obstacles are divided into two categories: static obstacles and dynamic obstacles. The positions of static obstacles are time-invariant and can be known in advance globally. Static obstacles mainly include islands, reefs, no-go areas, as well as shorelines in coastal waters or inland waterways, and underwater obstacles such as shipwrecks. The collision avoidance zone for static obstacles needs to expand the obstacle area outward by a certain safety margin to provide redundancy. Dynamic obstacles are usually other navigating vessels, whose positions, speeds, and headings change over time. Collision avoidance with dynamic obstacles requires predicting the motion of target vessels and the applicability of COLREGs rules. In most existing dynamic obstacle avoidance studies, the movement of target vessels is simplified to linear motion with no significant changes in speed or heading. Some studies consider collision avoidance actions compliant with COLREGs based on the relative motion status when a target vessel approaches the own ship. The basic navigation rules for two-vessel encounters are shown in Figure 4.

Ship domain plays a key role in collision avoidance decision-making. A ship domain is a structured safety buffer zone around a vessel [14], providing a spatial reference for collision avoidance behaviors. Obstacles are not allowed to enter the ship domain defined around the own ship. Building on the work of Zhou et al. [15], Chen et al. [16] constructed a dynamic dual-ship domain model, which can be expressed as:

(3) $$\left\{\begin{array}{cc} \mathrm{Action}\;\mathrm{domain}, & r_{\mathrm{safe}}<D\leq \min\left(R_{\mathrm{fore}},R_{\mathrm{aft}},R_{\mathrm{starb}},R_{\mathrm{port}}\right)\\ \mathrm{Safety}\;\mathrm{domain}, & r_{\mathrm{safe}}=2L\leq D \end{array}\right.$$

(4) $$\left\{\begin{array}{c} R_{fore}=L+\left(1+s\left(i\right)\right)\cdot T_{90}\cdot V_{0}\\ R_{aft}=L+T_{90}\cdot V_{0}\\ R_{starb}=B+DT\left(1+t\left(i\right)\right)\\ R_{port}=B+0.75DT\left(1+t\left(i\right)\right) \end{array}\right.$$

(5) $$T_{90}\approx \left(0.67/V_{0}\right)\cdot \sqrt{AD^{2}+{\left(DT/2\right)}^{2}}$$

$\left(R_{\mathrm{fore}},R_{\mathrm{aft}},R_{\mathrm{starb}},R_{\mathrm{port}}\right)$ are the radii of the action domain in the four directions, where *L* and *B* denote the length and breadth of the ship. AD is the advance, defined as the longitudinal distance traveled by the ship’s center of gravity from the moment the rudder is applied until the heading changes by 90°; DT is the tactical diameter, referring to the transverse offset of the ship’s center of gravity from the start of rudder action until the heading changes by 180°; $T_{90}$ represents the time required for the ship to complete a 90° heading change; s(i) and t(i) are coefficients corresponding to different encounter scenarios; and $V_{0}$ is the speed of the own ship. Their detailed calculation includes scaling rules for the radii, geometric relationships during encounters, relative speed, relative heading, and the vessel’s maneuverability parameters. The action domain provides a precautionary buffer for the safety domain. When entry of a target ship into this domain, COLREGs-compliant collision avoidance decisions shall be initiated. The size of the safety domain can be determined by the vessel’s stopping distance, which is typically approximated as twice the vessel length, i.e., 2L.

Factors affecting collision risk include the Distance at Closest Point of Approach (DCPA) and the Time to Closest Point of Approach (TCPA) [17]. Collision risk can be quantified using DCPA and TCPA, which constitutes the core idea of the velocity obstacle method adopted by Zhu et al. [18] for dynamic collision avoidance, and the basis for Zhang et al. [19]’s calculation of the dynamic collision risk index. These metrics can be calculated via Equations (6) and (7) [20]:

(6) $$\mathrm{DCPA}=S_{ot}\sin\left(\varphi_{r}-\theta -\pi \right)$$

(7) $$\mathrm{TCPA}=\frac{S_{ot}\cos\left(\varphi_{r}-\theta -\pi \right)}{v_{r}}$$

where $S_{ot}$ denotes the distance between the Own Ship (OS) and the Target Ship (TS), θ is the actual bearing angle between the two vessels, $\varphi_{r}$ is the relative heading of the TS with respect to the OS, and $v_{r}$ is the relative speed between the two vessels.

In addition to DCPA and TCPA, other risk indices can be used, such as the Ship Domain Operating Index (SDOI), Distance Closeness Index (DCI), and Operational Maneuvering Space Assessment (OMSA). In practical applications, to ensure the generalizability of risk calculations, it is usually necessary to consider the weights and normalization of different terms.

### 2.5. Path Optimization

Path optimization refers to multi-objective optimization and smoothing of an existing path based on basic voyage metrics. The fundamental metric for path optimization is voyage distance, and its calculation in existing studies is divided into three categories: Euclidean distance, great-circle distance, and rhumb-line distance. Euclidean distance is the straight-line distance on a plane, which is simple to calculate, highly efficient, and compatible with all path planning algorithms. The great-circle distance is the shortest distance between two points on Earth’s surface, which conforms to real navigation environments, but its disadvantage is that the heading needs to change more frequently. The rhumb-line distance yields a relatively longer voyage but requires fewer heading changes, which aligns with the characteristics of underactuated vessels. Euclidean distance is widely used in constrained waters or short-distance local path planning, but it suffers from significant errors in long-distance navigation. In contrast, great-circle distance and rhumb-line distance are more suitable for open waters or long-distance global path planning.

In addition, more research has shifted from the shortest path to multi-objective optimization, with the most frequently studied optimization objectives including minimum fuel consumption, shortest voyage time, highest comprehensive safety, and ease of maneuvering. Multi-objective optimization follows the Pareto principle; there is usually no absolute optimal solution (i.e., a dominated solution) that outperforms all other paths in every metric. The multi-objective path planning problem is NP-hard [21], and introducing multi-objective optimization significantly increases the parameter scale and the complexity of the solution space. In traditional manned vessels, multi-objective optimization is subject to subjective preferences; in ASV path planning, weights for different objectives need to be considered to obtain a satisfactory solution within an acceptable range.

Raw paths generated by graph-based path planning algorithms and sampling-based path planning algorithms often contain redundant waypoints, resulting in abrupt heading changes at these points that do not conform to vessel motion characteristics. Therefore, path smoothing is required. Commonly used path smoothing algorithms include the Douglas–Peucker algorithm, which simplifies waypoints while preserving the main features of the path; Bézier curves and B-spline curves, which smooth the path into continuous curves. These path smoothing algorithms further shorten the voyage and make the path more consistent with vessel motion characteristics. Fu et al. [22] employed a Dubins action set to generate paths satisfying both kinematic and temporal constraints. By introducing the Dubins action set as a replacement for a complex kinematic model, this method substantially reduces algorithmic complexity while retaining the ability to produce smooth, practically applicable paths. However, the cost is an increase in path length compared to other smoothing methods.

## 3. ASV Path Planning Algorithms

Path planning algorithms can be divided into four categories according to their core underlying ideas: graph-based path planning algorithms, sampling-based algorithms, numerical optimization-based algorithms, and artificial intelligence-based algorithms. The four types of algorithms have significant differences in the exploration mechanism in the path planning stage, and exhibit different characteristics in terms of path quality, solution efficiency, algorithm complexity, scalability, and extensibility.

Table 1 summarizes representative works from the four algorithm categories. These studies introduce improvements to the core path planning algorithms to varying degrees, achieving significant enhancements in functionality or performance. It should be noted that some studies only address dynamic obstacle avoidance without explicitly addressing static obstacles. Given that a dynamic obstacle with zero velocity can be regarded as a static obstacle, the capability of dynamic obstacle avoidance logically encompasses static obstacle avoidance. Therefore, for such studies, the static obstacle avoidance function is uniformly marked as “Y” in the table.

### 3.1. Graph-Based Path Planning Algorithms

Graph-based path planning algorithms model the navigation environment as a discrete graph structure, which uses nodes and edges to represent navigable areas, obstacle areas, and costs. Such algorithms rely on grid maps and have a controllable search process, making them the most mature algorithms in current path planning. Representative algorithms are Dijkstra’s algorithm, the A* algorithm, and their various variants [41].

Dijkstra’s algorithm was first proposed in 1959 [42]. Starting from the starting point, Dijkstra’s algorithm expands nodes according to the minimum cumulative cost without using a heuristic function to guide the search, which requires traversing a large number of nodes; thus, the search efficiency is low in large-scale maps. Zhou et al. [23] used an improved Dijkstra’s algorithm for global path planning, adopted the great-circle distance, and calculated the actual navigation time combined with environmental impacts to replace the traditional cost function represented by Euclidean distance. The influence of water depth on vessel passage was also considered, which is more suitable for the real navigation environment. Finally, the Douglas-Peucker algorithm was used to eliminate redundant waypoints and improve path smoothness. Zhen et al. [43] used a third-order Bezier curve was used to smooth the path after generation, further meeting the requirements of continuity for ship maneuvering. Borkowski et al. [44] presented a novel interpolation method for ship state prediction, which combined with a modified Dijkstra algorithm achieves dynamic collision avoidance, but it depends on pre-established accurate sea trial data.

The A* algorithm introduces a heuristic function on the basis of Dijkstra’s algorithm, and the total cost function given by [45]:

(8) $$F_{\mathrm{total}}\left(n\right)=g\left(n\right)+h\left(n\right)$$

where $F_{\mathrm{total}}\left(n\right)$ is the total estimated cost of node *n*; g(n) is the actual cost that has been incurred; and h(n) is the heuristic function, representing the estimated remaining cost from the current node to the target node. When the heuristic function is admissible (i.e., it never overestimates the true cost to reach the target), the optimality of the algorithm is guaranteed.

To address the limitations of the traditional A* algorithm in search direction, adaptability to dynamic obstacle avoidance, and multi-objective optimization, numerous studies have proposed improved schemes. Xu et al. [20] combined the characteristics of inland waterway channels and used the Density-Based Spatial Clustering of Applications with Noise (DBSCAN) algorithm to extract key turning points in the channel, then performed path planning segmentally using an improved A* algorithm. Compared with the four-direction search of the traditional A* algorithm, the improved A* algorithm was extended to sixteen-direction search, which greatly improved the smoothness of the initial path. Meanwhile, combined with a risk assessment model, the Dynamic Window Approach (DWA) was locally adopted to realize dynamic obstacle avoidance. Bienkowski et al. [46] integrated multi-objective optimization with the A* algorithm and proposed the New Approach to Multi-objective A* (NAMOA*) algorithm. They used the quadtree decomposition method for map modeling, combined with Dijkstra’s algorithm to find single-objective optimal solutions, and constructed a heuristic function using these solutions, enabling the NAMOA* algorithm to output a set of Pareto-optimal paths, effectively solving the trade-off among 12 objectives such as navigation distance and fuel consumption. In recent path planning research, the A* algorithm has been further developed, inspiring improvements for ASV applications. For example, Jiang et al. [47] used the spatio-temporal A* algorithm to solve the collision avoidance problem among multiple agents. Zhang et al. [48] proposed a constrained multi-layer bidirectional adaptive A* algorithm, whose cost function was not limited to path length, and sector search was used instead of traditional omnidirectional search to satisfy physical motion constraints. The algorithm efficiency was improved by maintaining bidirectional lists from the start to the target and from the target to the start. A dynamic step size was adopted, and virtual circles were used to ensure that the path did not pass through forbidden zones, improving path safety.

The D* algorithm is an improvement of the A* algorithm for dynamic environments. When the environment changes, the D* algorithm does not require full recalculation and significantly improves the response speed by incrementally updating the path. Chen et al. [16] proposed an improved D* algorithm integrated with a dynamic Dual Ship Domain (DSD*), which adopted 24-neighborhood search to make ship steering smoother, and combined the dual-ship domain model and COLREGs to realize compliant decision-making for dynamic collision avoidance.

The Theta* algorithm is an omnidirectional search variant of the A* algorithm that supports arbitrary-angle path generation, which was formalized by Daniel et al. in 2010 [49]. Liu et al. [24] proposed the non-uniform Theta* algorithm and the dynamic Theta* algorithm, in which the grid cost in the map dynamically changes with the distance to obstacles. Reverse search technology was adopted to avoid recalculation during path search and improve efficiency. Through the fusion of the two algorithms, the Theta* algorithm can generate paths that satisfy the COLREGs collision avoidance rules in narrow waters.

### 3.2. Sampling-Based Path Planning Algorithms

Sampling-based algorithms do not require precise discrete modeling of the environment. In essence, they extract a finite number of representative samples (candidate solutions) from an infinite or extremely large feasible solution space, without traversing all possible solutions, and approximate the optimum via sampled solutions. Sampling-based algorithms can rapidly generate near-optimal paths in complex unstructured environments, making them important techniques for realizing local collision avoidance and path planning in complex scenarios [50].

The main representative algorithm among sampling-based methods is the RRT* algorithm. The RRT* algorithm is an improvement over the Rapidly-exploring Random Trees (RRT) algorithm, and achieves asymptotic optimality by searching for the optimal parent node and performing rewiring. However, the traditional RRT* algorithm adopts random sampling, resulting in non-smooth paths that violate the kinematic constraints of ships. To address these limitations, many scholars have carried out targeted improvements.

Hu et al. [25] proposed a heuristic and adaptive RRT (HA-RRT) algorithm, which integrates the heuristic function from the A* algorithm into the RRT framework to guide node sampling, replacing the fully random sampling in the original RRT. A dynamic elliptic sampling domain was designed to reduce invalid sampling. Finally, a third-order Bezier curve was used to smooth the generated path. Meng et al. [26] proposed a circular sampling RRT* algorithm, which draws a circular domain centered on each sampling point and performs overlap detection with circular domains of existing sampling points, thus reducing redundant nodes in the sampling stage and improving sampling efficiency. Tao et al. [51] proposed a variable probability sampling RRT* intelligent algorithm combined with APF (VPS-APF-RRT*), which uses a dynamic bias rate to escape from local optima. The sampling direction is jointly determined by the resultant force calculated by the Artificial Potential Field (APF) algorithm and the original sampling direction of RRT*. When sampling encounters obstacles, critical points along the connecting line are found for connection instead of directly discarding samples, improving planning success in complex environments. Pan et al. [52] proposed the Dynamic Quickly RRT* (DQ-RRT*) algorithm, which dynamically adjusts the sampling step according to obstacle density, and introduces strategies such as obstacle-guided sampling, dynamic bias, and sparse sampling (discarding samples in densely sampled regions), enabling RRT* to be applied in dense obstacle environments. Finally, the bisection method and the triangle inequality are used to further refine the path, making it fit obstacles more closely and shortening the resulting path.

The above RRT*-based algorithms do not consider ship motion constraints, and the generated paths often suffer from abrupt heading changes. Some scholars have introduced Dubins curves into sampling algorithms to construct a Dubins motion set that conforms to ship maneuvering characteristics. Zhang et al. [53] proposed a position-based Dubins-RRT* algorithm, which introduces the Dubins action set into path planning. The travel between waypoints except the destination is simplified as a relaxed Dubins problem, and bias probability is added to balance motion constraints and algorithm efficiency. Zhang et al. [27] introduced a fuel consumption-aware heuristic function on the basis of the position-based Dubins-RRT* algorithm, and proposed the heuristic position-based Dubins-RRT* (HP-Dubins-RRT*) algorithm. In polar water environments, a non-uniform probabilistic sampling field is constructed according to resistance to reduce the high sampling variance of RRT*.

In addition to the RRT family of algorithms, the isochrone method is also an important type of sampling-based algorithm. The core idea of the algorithm is to construct the ship’s reachable region with a fixed time step, where the sampling step adapts to the real-time ship speed. Ha et al. [28] applied an improved isochrone method to path planning with quantified collision risk, achieving satisfactory safety in multi-ship encounter scenarios.

### 3.3. Optimization-Based Path Planning Algorithms

Numerical optimization-based path planning algorithms transform the path planning problem into a constrained nonlinear optimization problem [41], which is typically solved through iterative minimization of the objective function. Such methods do not require discretized environmental modeling, can handle multi-constraint problems in continuous space, and are capable of generating smooth continuous paths.

The Fast Marching Method (FMM) was first proposed by Adalsteinsson and Sethian in 1995 [54]. The core of FMM is to describe wave propagation via the Eikonal equation, but it assumes isotropic propagation, meaning that waves spread at the same speed in all directions, which cannot reflect the anisotropic effects of ocean currents and wind fields on ship navigation in the marine environment. To address this drawback, Zhang et al. [29] used an improved Anisotropic Fast Marching (AFM) algorithm, wherein static obstacles, channel boundaries, and dynamic environmental fields were fused into a composite guidance field for anisotropic wavefront expansion, thereby enabling path planning in restricted bridge-area waters.

The Possible Point of Collision (PPC) algorithm constructs safe paths by identifying potential collision points and generating avoidance waypoints. Lazarowska [30] proposed a cascade search strategy based on the PPC algorithm to identify vessels with the highest collision probability, generate waypoints complying with COLREGs around them, and expand step by step until a complete safe path is formed.

Eskandari et al. [55] formulated path planning as a Model Predictive Control (MPC) optimization problem. Cooperative localization and communication constraints were realized by constructing a consensus graph. To address the non-convex and NP-hard characteristics of the problem, sequential convex programming was adopted to decompose the original problem into multiple subproblems for convex approximation, thereby enabling cooperative path planning for ASVs and autonomous underwater vehicles (AUVs).

Chen et al. [32] applied the parameterized level-set method to solve multi-objective optimization problems. By improving the edge detection function and adopting distance regularization, dynamic environmental factors such as ocean currents and wind fields were integrated into the level-set evolution process, achieving multi-objective optimization in a dynamic marine environment. However, the vessel was modeled as a point mass, without considering its kinematic or dynamic characteristics.

The trajectory homotopy algorithm uses heuristic rules to prune the path set, reducing the infinite path search space to an extremely small subset, thereby transforming the complex mathematical problem of path planning into a practical engineering problem. Building on the concept of trajectory homotopy, Yang et al. [56] proposed the Pseudo-Trajectory Homotopy Method (PTHM), which adopts a fixed “spiral motion + parabolic motion” splicing template to generate diving and surfacing trajectories for AUVs. By adjusting only the pitch angle parameters to generate different candidate solutions, the algorithm can simultaneously satisfy the kinematic constraints and diving-surfacing efficiency requirements of AUVs. Path planning for both AUVs and ASVs shares the common challenges of kinodynamic constraints and an infinite continuous path space. Therefore, the engineering design philosophy of PTHM, characterized by “parameterized template + heuristic pruning”, holds important reference value for solving ASV path planning problems.

Heuristic intelligent optimization algorithms search for optimal solutions by simulating natural phenomena or biological behaviors, and exhibit strong robustness in handling non-convex and NP-hard problems. Typical algorithms include genetic algorithms, ant colony optimization, and other evolutionary algorithms. Wang et al. [31] used the A* algorithm to generate an initial single-objective optimal solution, and then employed the third-generation Non-Dominated Sorting Genetic Algorithm (NSGA-III) to output a Pareto solution set with comprehensive optimality for voyage distance, fuel consumption, and other objectives, addressing the multi-objective routing problem in polar navigation environments. Huang et al. [57] proposed a hybrid deep learning and dynamic ant colony algorithm, which improved the pheromone update rule with time-varying evaporation, elite enhancement, and simulated annealing Cauchy heavy-tailed perturbation. Deep learning was used for adaptive parameter tuning, significantly improving planning efficiency and robustness in complex environments.

### 3.4. AI-Based Path Planning Algorithms

The emergence of artificial intelligence technologies has provided new solutions for ASV path planning. Such algorithms can autonomously learn complex decision-making strategies. In current research, artificial intelligence techniques in the field of ASV path planning are mainly used to process high-dimensional historical state data and realize autonomous path planning.

#### 3.4.1. Processing High-Dimensional Historical State Data

The mainstream algorithms adopted in current research are the Long Short-Term Memory (LSTM) network and its variants. Through the gating mechanisms including input gate, forget gate, and output gate, LSTM can effectively capture the temporal characteristics of ship motion and historical navigation habits. When applied to the own ship, they can smooth the own ship’s maneuvers, enhance decision stability, and reduce abrupt or unnecessary steering actions. For example, Waltz et al. [33] used LSTM to equip path planning algorithms with temporal information processing capability, thereby improving the stability of decision-making. Wang et al. [40] employed LSTM to retain key historical states, making action evaluation more empirical.

On the other hand, LSTM can achieve high-precision trajectory prediction. Accurate prediction of the motion trajectory of dynamic obstacles is a prerequisite for dynamic collision avoidance decision-making. In traditional algorithms, the motion of target ships is usually simplified as uniform linear motion. Therefore, the planner must recompute the trajectory when the observed state of a target ship changes, and the safety margin for dynamic obstacle avoidance is insufficient. For instance, Song et al. [58] extracted human driving preferences from AIS data based on LSTM networks combined with the K-means algorithm, realizing ship trajectory prediction that is more consistent with human maneuvering habits. Finally, collision avoidance decisions were completed using techniques such as knowledge graphs and the DWA algorithm. Suo et al. [59] employed a Gate Recurrent Unit (GRU) model for ship trajectory prediction. Compared with LSTM, GRU merges the forget and input gates into a single update gate, thereby reducing parameter count and computational overhead while maintaining comparable short-term prediction accuracy. Chen et al. [60] developed a ship trajectory prediction model based on the Crossformer architecture, which achieves significantly lower errors in both latitude and longitude prediction than traditional LSTM-based methods, leading to more accurate trajectory prediction.

#### 3.4.2. Path Planning

Path planning is a sequential decision-making problem, while reinforcement learning (RL) enables agents to learn decisions through reward feedback via interaction with the environment; the two are highly compatible. With the development of deep neural networks, Deep Reinforcement Learning (DRL), which combines the perception ability of deep learning and the decision-making ability of reinforcement learning, has become a research hotspot for autonomous path planning of ASVs. The characteristics of reinforcement learning are shown in Table 2.

Early reinforcement learning is suitable for simple scenarios with discrete states (e.g., grid maps) and action spaces. The Q-Learning algorithm [61] stores state-action values via tables, featuring simple implementation and fast and stable convergence. For instance, Silva Jr. et al. [34] applied Q-Learning to sailboat path planning, wherein an initial 8-direction action set is dynamically adjusted according to wind direction, retaining only feasible navigation headings. As an on-policy temporal difference algorithm, SARSA [68] strictly binds policy evaluation and policy execution, leading to more conservative updates than Q-Learning and better adaptability to safety constraints. The Monte Carlo policy gradient algorithm (REINFORCE) [62] is a policy-based RL method that updates parameters after an episode is completed, directly optimizing the policy itself and supporting continuous action spaces, but it suffers from large variance and unstable convergence.

Traditional reinforcement learning cannot handle high-dimensional inputs or adapt to rapidly developing perception technologies (e.g., visual images). Deep Reinforcement Learning (DRL) combines the advantages of deep learning and reinforcement learning, using deep neural networks to fit value functions or policies, thus overcoming this limitation.

Deep Q-Network (DQN) combines Convolutional Neural Network (CNN) with Q-Learning and introduces experience replay and target networks, overcoming the limitation that traditional tabular algorithms cannot handle high-dimensional continuous states. However, it only supports discrete action spaces and suffers from Q-value overestimation. In recent studies, Hasselt et al. [69] proposed Double DQN, which decouples action selection from action evaluation, greatly improving policy stability. Wang et al. [70] split the Q-network into state value and action advantage, proposing Dueling DQN, which improves sample efficiency and adapts to sparse reward environments. For path planning applications, Lee et al. [35] used DQN to accomplish ship path planning between ports. Eight-direction exploration was performed on grid maps, comprehensively considering environmental impacts on navigation safety and existing waterways. The ε-greedy strategy was adopted to balance exploration and exploitation. Finally, the Douglas–Peucker algorithm was used to further smooth the path. Pan et al. [36] proposed an enhanced DQN suitable for multi-objective optimization and multi-ship collision avoidance. Based on traditional DQN, it introduces dueling architecture to separate state value and action advantage estimation, and uses a double Q-network to alleviate Q-value overestimation.

The Actor-Critic (AC) framework separates decision-making and evaluation, significantly reducing policy gradient variance. In the AC framework, the Actor network outputs actions and the Critic network evaluates action values, supporting continuous action spaces. It has become the dominant DRL framework for ASV path planning.

Deep Deterministic Policy Gradient (DDPG) outputs deterministic continuous policies, solving the problem that traditional RL cannot handle continuous actions [65]. However, its single critic network tends to cause Q-value overestimation. Twin Delayed DDPG (TD3) [71] improves training stability by using double critic networks, delayed policy updates, and target policy smoothing. Xu et al. [37] proposed Knowledge Transfer DDPG (KTDDPG), which integrates knowledge transfer to realize high-quality COLREGs-compliant sample transfer, greatly improving convergence speed and collision avoidance success rate. Yin et al. [72] used multi-agent DDPG (MADDPG) for multi-ship path planning, characterized by centralized training and decentralized execution, and incorporated LSTM to analyze historical data, reducing the search space and improving MADDPG efficiency.

Trust Region Policy Optimization (TRPO) [73] strictly limits policy update range via trust region constraints, solving the problems of unstable training and easy collapse in traditional policy gradient methods. Proximal Policy Optimization (PPO) simplifies TRPO by introducing a clipped surrogate objective, which restricts policy updates without requiring second-order optimization, making training more stable [66].

Soft Actor-Critic (SAC) is an off-policy DRL algorithm based on maximum entropy reinforcement learning, whose core is to balance exploration and exploitation through entropy regularization [67]. SAC also features off-policy training and stochastic policy output, providing stronger generalization and maintaining excellent performance in unknown dynamic environments. Zhao et al. [38] replaced the Gaussian policy in SAC with Hamiltonian Monte Carlo sampling and introduced a new leapfrog operator to improve exploration efficiency and sampling stability. Prioritized experience replay was adopted to enhance learning efficiency. Wang et al. [40] integrated LSTM with SAC, using LSTM to reduce the dimension of historical state data, enabling SAC to make action decisions more consistent with the underactuated characteristics of ships. Yu et al. [39] constructed a dynamic state space using the K-means clustering algorithm based on SAC to identify collision risks more accurately. COLREGs compliance items and dynamic risk items were added to the reward function design. The algorithm maintained excellent collision avoidance performance even in environments with dynamically changing numbers of obstacle ships.

The AC framework updates network parameters through the TD error. The common one-step TD error is expressed as:

(9) $$\delta =\left(r+\gamma V(s^{\prime })\right)-V\left(s\right)$$

where *r* is the immediate reward, γ is the discount factor, *V* is the state value function, *s* is the current state, and $s^{\prime }$ is the next state.

The SAC algorithm adopts dual Critic networks to mitigate Q-value overestimation, and its TD error is calculated as follows:

(10) $$y=r+\gamma \left(\min_{i=1,2}Q_{\phi_{i}}\left(s^{\prime },a^{\prime }\right)-\alpha \log\pi_{\theta }\left(a^{\prime }|s^{\prime }\right)\right)$$

(11) $$\delta =Q_{\phi }\left(s,a\right)-y$$

where $a^{\prime }$ is the sampled action, *Q* is the output value of the Critic network, α is the temperature coefficient of SAC, and $-\log\pi_{\theta }\left(a^{\prime }|s^{\prime }\right)$ is the policy entropy of SAC, which encourages stochastic exploration.

Most mainstream deep reinforcement learning algorithms at present are off-policy algorithms. On-policy algorithms use real-time data generated by the current policy to update the policy; off-policy algorithms can utilize historical data from other policies and experience replay buffers. This means that on-policy algorithms have lower experience utilization efficiency than off-policy algorithms in multi-agent environments and require more training episodes. However, on-policy algorithms are not affected by historical data, thus exhibiting higher training stability than off-policy algorithms.

Action spaces are divided into two categories: discrete and continuous. Discrete action spaces reduce the difficulty of model training but do not conform to real-world ship motion control. Although continuous action spaces make model training more challenging, the almost non-repetitive sampling of continuous actions reduces the risk of overfitting.

In addition to the above applications, deep learning-based object detection algorithms such as Faster Regions with Convolutional Neural Network (Faster R-CNN), You Only Look Once (YOLO) and Single Shot MultiBox Detector (SSD) have been extensively utilized for ship detection, obstacle recognition and situational awareness tasks [74]. Chen et al. [75] developed the unsupervised enhancement-low-light-image-network generative adversarial network (EnlightenGAN)-based EG-YOLO+ framework, which enhances low-light ship detection performance and provides stronger support for safe path planning of ASVs. In the execution phase of path planning, Gao et al. [76] presented an event-driven prescribed performance RL optimal anti-disturbance control scheme, greatly improving the reliability and cost-effectiveness of trajectory tracking under complex sea conditions. Moreover, large language models (LLMs) have been attempted to solve high-level task planning or ship encounter decision-making. Christensen et al. [77] used prompt engineering to parse ambiguous natural language into clear and executable action sequences, supporting task progress tracking and dynamic task replanning. Zheng et al. [78] constructed an LLM-based non-parametric model to simulate human cognition of COLREGs and encounter decision-making, and introduced a verification and correction module to reduce the impact of LLM hallucinations. The application of LLMs in path planning is still in its infancy, and there remain many challenges. First, they cannot output deterministic optimal solutions. Second, LLM hallucinations remain inherently difficult to mitigate. Furthermore, the inference process of LLMs is a black box, leading to poor interpretability. Finally, the definition of accident liability is unclear. Relevant technologies still require further research and breakthroughs.

## 4. Algorithm Analysis

There is a wide variety of path planning algorithms, including adaptive improvements to traditional graph search, sampling, and numerical optimization algorithms for ship path planning, as well as innovative integrated applications of artificial intelligence technologies. Based on different solution ideas and technical frameworks, these algorithms exhibit significant differences in applicable scenarios, performance, and engineering application value. ASV path planning is not an isolated mathematical problem, but a core decision-making component of modern autonomous ship navigation systems. Therefore, algorithm performance needs to be comprehensively evaluated from a systems engineering perspective. Algorithms must not only generate high-quality feasible paths, but also meet the requirements of real-time performance, scalability, extensibility, and engineering applicability. This chapter systematically compares and analyzes the four mainstream categories of algorithms from three dimensions: path quality generated by the algorithm, algorithm scalability and extensibility, and performance. Table 3 provides a structured comparison of the four algorithm categories across the three dimensions discussed in this section.

### 4.1. Path Quality

The quality of the generated path is a core indicator for evaluating the performance of ASV path planning algorithms, directly determining the safety, economy, and executability of navigation. Path quality can be evaluated from two dimensions: collision avoidance quality and optimization quality.

Collision avoidance is the primary safety constraint, and its quality is reflected in two aspects: the success rate of collision avoidance and compliance with COLREGs. Since the positions of static obstacles are fixed and globally known, all types of planning algorithms can achieve efficient static obstacle avoidance. For example, the improved RRT* algorithm proposed by Meng et al. [26] and most DRL algorithms maintain excellent collision avoidance performance in complex static obstacle environments, while Devo et al. [79] used deep reinforcement learning to solve 3D maze navigation. At present, static obstacle avoidance technology is relatively mature and no longer constitutes a limiting factor in ASV path planning. In dynamic obstacle avoidance, the positions, speeds, and headings of dynamic obstacles change over time, which is a key challenge for collision avoidance decision-making. Traditional algorithms often require significant modifications or combinations with other algorithms to achieve this goal. Xu et al. [20] realized dynamic obstacle avoidance by combining the DWA algorithm, and the improved spatio-temporal A* algorithm proposed by Jiang et al. [47] relaxed the grid-based discretization constraints to a certain extent. Lazarowska [30] achieved collision avoidance by iteratively solving avoidance paths for potential collision points, but the computational complexity was relatively high. By contrast, artificial intelligence algorithms perform better in handling dynamic obstacle avoidance. Zhao et al. [38], Yu et al. [39], and Pan et al. [36] all realized collision avoidance decision-making in dynamic obstacle environments, and Pan et al. [36] and Xu et al. [37] achieved COLREGs-compliant collision avoidance decisions. Furthermore, Waltz et al. [33] used LSTM to predict the trajectories of target ships, further improving collision avoidance success rates.

The optimization quality of the path requires consideration of multi-objective optimization problems for multiple navigation indicators, with path length and path smoothness as basic indicators. For example, graph-based path planning algorithms such as Dijkstra’s algorithm and A* algorithm can guarantee the shortest path. Sampling algorithms based on RRT* guarantee asymptotic optimality. Numerical optimization algorithms and artificial intelligence algorithms, in terms of path length, demand substantial computational resources or extensive training to approach the optimal solution. Regarding path smoothness, Zhou et al. [23], Zhen et al. [43], Hu et al. [25], and other scholars applied additional path smoothing techniques. Common path smoothing algorithms include the Douglas–Peucker algorithm and Bézier curves. Lyu et al. [80] introduced the Random-Escape Particle Swarm Optimization (Random-Escape PSO) algorithm to further eliminate redundant waypoints of the A* algorithm and improve waypoint safety based on Bézier curve optimization. Instead of smoothing the generated path, Chen et al. [16] and Wang et al. [40] chose to optimize the exploration or action space to promote inherent path smoothness during the generation process. Owing to their different action spaces, AI-based algorithms exhibit varying degrees of path smoothness. In terms of path smoothness, discrete action space algorithms are comparable to their traditional counterparts. Conversely, for continuous action space algorithms, the resulting path smoothness approaches that of optimization-based methods. In summary, there are many path smoothing methods, but when smoothing a generated path, it is difficult to fully embed the vessel’s kinematic constraints, and instead the turning points are adjusted to meet ship performance by minimizing the frequency of course alterations. Incorporating ship kinematic constraints during path generation to ensure the path is generated within the performance range of the ship is the current development trend.

Optimization of additional navigational metrics beyond path length and smoothness follows the Pareto optimality principle. Existing optimization methods are divided into two categories. Bienkowski et al. [46] and Wang et al. [31] first solved single-objective optimal subproblems for each indicator, and then calculated the Pareto optimal solution set for multi-objective path planning. Yin et al. [72] and Yu et al. [39], on the other hand, chose to incorporate multiple navigation indicators into the reward function design and used artificial intelligence algorithms to solve multi-objective optimization problems in path planning.

### 4.2. Scalability and Extensibility

#### 4.2.1. Scalability

Good scalability requires that an algorithm maintains stable computational efficiency and performance under scenarios involving increased map scale, higher obstacle density and complexity, and multi-ship collaborative navigation.

The core of multi-agent cooperative planning is to resolve multi-ship conflict elimination and task allocation. Extending traditional algorithms from single-agent to multi-agent scenarios requires the introduction of additional complex mechanisms, such as centralized task allocation, conflict resolution, and game theory. For example, Wen et al. [81] and Jiang et al. [47] introduced a binary conflict tree combined with an improved A* algorithm to solve conflicts among multiple agents. By contrast, artificial intelligence-based algorithms are inherently better suited to multi-agent coordination. For instance, Yin et al. [72] adopted a multi-agent reinforcement learning algorithm with a centralized training and decentralized execution framework, which can realize multi-ship cooperative decision-making through a shared experience pool without additional complex conflict resolution mechanisms.

#### 4.2.2. Extensibility

Excellent extensibility demands that the algorithm can incorporate new constraints, integrate functional modules, or be applied to new navigation scenarios without requiring large-scale reconstruction of its core framework or fundamental changes to its core algorithmic logic.

For graph-based path planning algorithms, adding constraints requires reconstructing the cost function and heuristic rules. Moreover, to ensure a globally optimal path, the costs in all grid cells of the map must be recalculated, resulting in considerable restructuring effort. For example, Chen et al. [16] expanded the search neighborhood to 24-neighborhood to adapt to COLREGs and reconstructed the node expansion logic. For sampling-based path planning algorithms, new constraints are mainly implemented by modifying the sampling probability distribution and collision detection rules. For instance, when considering the underactuated characteristics of ships in the RRT* algorithm, the introduction of the Dubins action set replaces straight-line collision detection between waypoints with wider-range avoidance detection, leading to moderate restructuring. Numerical optimization-based path planning algorithms can theoretically accommodate multiple constraints but suffer from limited engineering practical applicability. Examples include the improved level-set algorithm by Chen et al. [32] and the improved velocity obstacle method by Han et al. [82]. After adding new constraints, the non-convex and nonlinear characteristics of the problem must be considered to resolve issues such as iterative non-convergence and infeasible solutions, as well as to devise tractable approximations for NP-hard problems [83,84], leading to significantly increased algorithm complexity. Artificial intelligence-based algorithms exhibit the greatest extensibility with respect to new constraints. New constraints can be implemented through three approaches: state space expansion, embedding weighted terms in the reward function, and action space clipping. Since the state space, reward function, and action space are inherently extensible, no restructuring of the core algorithm framework is required. For example, Xu et al. [37] introduced knowledge transfer to enable the algorithm to quickly adapt to new constraints. With respect to control system integration, through optimized design of the action space, deep reinforcement learning algorithms with continuous action spaces can output control commands such as heading and thrust, making them compatible with mainstream controllers such as PID.

When integrating the motion control module into the algorithm, the focus is mainly on whether the path satisfies ship dynamics and maneuvering constraints. Most traditional path planning algorithms output discrete waypoint sequences containing only position information. Even when the Dubins action set is considered, intermediate waypoints still ignore heading information to ensure efficiency. Therefore, paths generated by traditional algorithms require further processing via trajectory tracking algorithms. Artificial intelligence-based algorithms have the ability to directly output continuous control commands. Deep reinforcement learning algorithms with continuous action spaces (such as DDPG, PPO, and SAC) can directly output control variables such as heading angle and thrust. Wang et al. [40] incorporated a ship motion control module to further verify the favorable compatibility of deep reinforcement learning in ship control.

In summary, artificial intelligence algorithms based on deep reinforcement learning show significant advantages in three dimensions: constraint expansion, multi-agent coordination, and interfacing with the control layer. However, compared with traditional algorithms, they lack interpretability (especially those incorporating LLMs), and may generate paths that are correct but incomprehensible to humans, thereby reducing the human operators’ trust in the system. Furthermore, AI-based algorithms cannot guarantee the success rate of planning after generalization, and when a planning failure occurs, it is difficult to rapidly pinpoint the source of the error.

### 4.3. Algorithm Performance

Algorithm performance is a key consideration for the transition of ASV path planning from theoretical research to engineering applications. It is mainly evaluated from two perspectives: computational efficiency and generalization ability after environmental changes. The former determines whether the algorithm can meet the requirements of real-time navigation path planning; the latter determines the reliability of the algorithm in unknown and time-varying marine environments.

Computational efficiency directly determines the speed of path generation and is the core guarantee for realizing real-time obstacle avoidance and dynamic replanning. For graph-based algorithms, the time cost is mainly concentrated on node expansion and cost calculation. The greater the number of search directions, the larger the search neighborhood, and the finer the grid resolution, the higher the computational complexity. For sampling-based algorithms, the time cost is dominated by random exploration and collision detection. Biased sampling, elliptical constraints, and sparsification strategies can improve convergence speed by reducing invalid sampling. However, the path quality of sampling-based algorithms depends on the number of samples, so improving path accuracy will significantly increase the time cost. Numerical optimization-based algorithms rely on iterative optimization, with high computational cost and unstable convergence speed. Especially under multi-constraint, non-convex, and nonlinear conditions, the number of iterations required for solving increases significantly. Artificial intelligence-based algorithms, especially deep reinforcement learning algorithms, have the characteristic of “slow training but fast inference”. The model training time is affected by network structure, reward function design, hyperparameters, and hardware performance. Algorithms using continuous action spaces require more training episodes to converge than those using discrete action spaces. In addition, cold start demands significantly more time than warm start. Nevertheless, the response speed during the inference stage can meet the replanning requirements in dynamic environments.

Good generalization ability enables the algorithm to still output feasible paths stably when the environment changes unexpectedly, which is crucial for ASVs to adapt to complex marine environments. For traditional graph-based, sampling-based, and numerical optimization-based algorithms, once the environmental model or constraints change, they require remapping, cost recalculation, or re-iteration. Their generalization relies on accurate prior maps, leading to weak adaptability to dynamic and unknown environments. Unlike traditional algorithms, artificial intelligence-based algorithms learn decision-making strategies through interaction with the environment. Provided that the training covers sufficient scenarios, such algorithms have stronger environmental adaptability. Moreover, robustness in new environments can be further improved through observation space normalization, transfer learning, and other techniques. Although deep reinforcement learning algorithms suffer from overfitting and underfitting, their generalization potential is significantly higher than that of traditional methods.

In summary, traditional algorithms have stable computational efficiency and stronger path optimality in static and known environments, making them suitable for global planning and static route design. Artificial intelligence algorithms have obvious advantages in generalization in dynamic environments and are more suitable for local real-time obstacle avoidance, dynamic replanning, and decision-making in complex encounter scenarios. For future path planning, a hybrid architecture can be considered, which uses traditional algorithms for global planning and artificial intelligence algorithms for local optimization, so as to balance path quality and cross-scenario generalization performance. The advantages, limitations, and applications of the four categories of ASV path planning algorithms are summarized in Table 4.

## 5. Conclusions and Future Outlook

This paper has systematically reviewed the research status of ASV path planning, and has classified existing path planning algorithms into four categories: graph-based, sampling-based, optimization-based, and artificial intelligence-based algorithms. The four types of algorithms have been compared and analyzed from three dimensions: path quality, scalability and extensibility, and algorithm performance.

Traditional graph-search and sampling-based algorithms exhibit limited scalability and extensibility in complex marine environments. Nevertheless, they remain competitive. This is attributed to their ability to generate high-quality paths in static environments, as well as the excellent computational efficiency achieved by their improved variants. Numerical optimization methods are prone to local minima and computational inefficiency as the number of objectives and constraints grows. Nevertheless, they can effectively handle multi-objective and nonlinear constraint problems, produce paths with superior smoothness, and perform favorably in low-dimensional spaces. In contrast to the outstanding performance of traditional algorithms in global path planning and static environments, deep reinforcement learning, as a representative AI-based algorithm, offers superior capabilities in dynamic decision-making, generalization, and integration with vessel motion control. The work of Liu et al. [41] further corroborates the following trends. First, AI-based techniques are being widely adopted. Second, hybrid algorithms show particularly strong promise: By integrating complementary strengths from different paradigms into a core planning framework, they achieve both stronger adaptability and superior optimization performance. Third, there is a growing emphasis on algorithmic practicality and regulatory compliance. In summary, ASV path planning is evolving from single-path feasibility toward multi-constraint, multi-objective optimization with autonomous decision-making in dynamic environments.

Significant progress has been made in the field of tracking control in recent years, which has promoted the translation of path planning algorithms from simulation to real-world deployment. For example, Zhu et al. [85] proposed the cooperative localization-formation hybrid framework, which achieves finite-time convergence of formation tracking errors to a small neighborhood around zero and reduces communication overhead in multi-USV systems, but high-accuracy robust tracking in complex marine conditions remains to be improved [86]. In addition, several critical challenges remain in practical engineering applications.

1. **Strengthening sim-to-real transfer techniques**: Bridging the gap between simulation and physical trials requires high-fidelity coupled modeling of complex marine environments. Time-varying disturbances—including wind, waves, and currents—must be explicitly incorporated into the cost function, and vessel motion characteristics must be tightly integrated into the planning algorithm. Constructing such realistic simulation environments is essential to minimize the risks associated with real vessel deployment.
2. **Real-world validation of COLREGs-compliant dynamic collision avoidance**: The verifiable integration of COLREGs into dynamic obstacle avoidance remains an open challenge. Future research must address multi-vessel conflict resolution with real-time decision-making, and these methods must be validated in real maritime scenarios rather than simulation.
3. **Onboard-optimized algorithm design**: Appropriately reducing algorithmic complexity to align with onboard computational capabilities is pivotal to achieving practical vessel deployment.

By reviewing and comparing ASV path planning algorithms, this paper aims to provide a reference for research on path planning technologies for ASV autonomous navigation and dynamic collision avoidance, and promote the further development of relevant theories and engineering applications.

## Figures and Tables

**Table 1:** Representative articles in each method.

Methods	Core Ideas	Ship Motion Model	Static Obstacle	Dynamic Obstacle	COLREGs Compliance
Graph-Based	Dijkstra [23]	Y	Y	Y	Y
	A * [20]	N	Y	Y	N
	D * [16]	Y	Y	Y	Y
	Theta * [24]	Y	Y	Y	Y
Sampling-Based	HA-RRT [25]	N	Y	N	N
	RRT * [26]	N	Y	N	N
	Dubins-RRT * [27]	Y	Y	N	N
	Isochrone [28]	Y	Y	Y	Y
Optimization-Based	AFM [29]	N	Y	N	N
	PPC [30]	Y	Y	Y	Y
	NSGA-III [31]	N	Y	N	N
	Level-Set [32]	N	Y	N	N
AI-Based	LSTM-TD3 [33]	Y	Y	Y	Y
	Q-Learning [34]	N	Y	N	N
	DQN [35]	N	Y	N	N
	Enhanced DQN [36]	N	Y	Y	Y
	KTDDPG [37]	Y	Y	Y	Y
	SAC [38,39]	Y	Y	Y	Y
	LSTM-SAC [40]	Y	Y	Y	N

* Y = Yes, N = No.

**Table 2:** Characteristics of reinforcement learning algorithms.

Algorithm	Category	Policy Type	Action Space
Q-Learning [61] (1992)	Value-Based	Off-Policy	Discrete
REINFORCE [62] (1992)	Policy-Based	On-Policy	Discrete/Continuous
SARSA [63] (1994)	Value-Based	On-Policy	Discrete
DQN [64] (2013)	Value-Based	Off-Policy	Discrete
DDPG [65] (2015)	Actor-Critic	Off-Policy	Continuous
PPO [66] (2017)	Actor-Critic	On-Policy	Discrete/Continuous
SAC [67] (2018)	Actor-Critic	Off-Policy	Continuous

**Table 3:** Comparison of four categories of ASV path planning algorithms.

Evaluation Dimension	Graph-Based	Sampling-Based	Optimization-Based	AI-Based (DRL)
Path Quality				
Static Collision Avoidance	High	High	High	High
Dynamic Collision Avoidance	Low	Low	Medium	High
COLREGs Compliance	Low	Low	Low	High
Path Optimality (e.g., Length)	High	Medium	High	Medium
Path Smoothness	Low	Low	High	Low (Disc.)
				High (Cont.)
Scalability & Extensibility				
Large-Scale Map Adaptability	Low	Medium	Low	High
Multi-Agent Coordination	Medium	Low	Medium	High
New Constraint Integration	Low	Medium	Low	High
Control Layer Compatibility	Low	Low	Medium	High
Algorithm Performance				
Computational Efficiency	Medium	High	High (Low-Dim.)	Low (Training)
				High (Inference)
Generalization Ability	Low	Low	Low	High
Engineering Landing Difficulty	Medium	Medium	Low	High

Note: Disc. = Discrete action space, Cont. = Continuous action space, Low-Dim. = Low-Dimensional.

**Table 4:** Summary of Four Categories of ASV path planning algorithms.

Evaluation Dimension	Graph-Based	Sampling-Based	Optimization-Based	AI-Based (DRL)
Core Advantages	Optimal path, mature	Fast in complex environments	Smooth path	Dynamic adaptation, generalization ability
Main Limitations	Poor dynamic adaptability	Non-smooth path	Local optimum	Slow training
Typical Applications	Global path planning	Local path planning	Path optimization	Dynamic collision avoidance

## Data Availability

No new data were created or analyzed in this study. Data sharing is not applicable to this article.
